# The Rising Threat of Antibiotic Resistance in Poultry: Veterinary and One Health Perspectives

**DOI:** 10.3390/vetsci12111059

**Published:** 2025-11-04

**Authors:** Shaikh Sumayya Sana, David Atuahene, Vivien Nagy, Ayaz Mukarram Shaikh, Renáta Knop

**Affiliations:** 1 Department of Animal Science, Institute of Animal Science, Faculty of Agricultural and Food Sciences and Environmental Management, University of Debrecen, 4032 Debrecen, Hungary; sumayya.sana@agr.unideb.hu; 2 School of Agriculture and Veterinary Medicine, University of Turin, Grugliasco, 10095 Turin, Italy; david.atuahene@unito.it; 3 Department of Planetary Health, One Health Institute, Faculty of Health Sciences, University of Debrecen, 4032 Debrecen, Hungary; nagy.vivien@agr.unideb.hu; 4 Faculty of Agriculture, Food Science and Environmental Management, Institute of Food Science, University of Debrecen, 4032 Debrecen, Hungary

**Keywords:** antibiotic resistance, poultry, One Health, zoonotic transmission, alternatives to antibiotics, food safety

## Abstract

**Simple Summary:**

Antibiotics have historically been employed in poultry to avert disease and promote growth; however, excessive usage has facilitated the emergence of resistant bacteria, jeopardizing the health of avians, humans, and the ecosystem. This review examines important scientific databases and synthesizes existing research regarding antibiotic resistance in poultry from a One Health viewpoint, integrating animal, human, and environmental health. We elucidate the mechanisms by which resistance emerges and disseminates from agricultural settings to the broader community, and we assess viable alternatives to antibiotics, such as probiotics, prebiotics, enzymes, and essential oils. We also emphasize tangible obstacles such as expenses, insufficient awareness, and inadequate coordination, that impede advancement. Poultry farmers can sustain production and decrease antibiotic usage by implementing evidence-based alternatives and effective management practices within a framework of supporting legislation. Enduring solutions necessitate collaborative efforts from governments, industry, veterinarians, researchers, and consumers to foster innovation, education, and equitable access to resources, thereby safeguarding animal welfare, public health, and food security.

**Abstract:**

The extensive application of antibiotics in poultry production has resulted in the emergence of resistant bacteria, which pose a great threat to the health of birds and humans. In this review, the literature is searched using databases such as PubMed, Scopus, Web of Science, and Google Scholar. Studies concerning antimicrobial resistance in poultry, the One Health approach, and alternative strategies to antibiotics are included, while studies not in English, opinion-based papers, and studies not related to poultry or AMR are excluded. This review explores the increasing challenges of antibiotic resistance in poultry, emphasizing the One Health framework related to animal, human, and environmental health. The risks of zoonotic transmission from poultry, the mode of development of resistance, and alternative antibiotics (comprising probiotics, prebiotics, enzymes, and essential oils) are the key topics discussed. This review further touches on critical barriers in fighting antibiotic resistance, which include economic constraints, a lack of awareness, and coordination challenges. This study highlights regulatory and consumer-driven changes in antibiotic use. The poultry industry can reduce the use of antibiotics by adopting the One Health approach and implementing evidence-based alternatives that support productivity. However, sustainable solutions require further research, policy reforms, and collaboration across sectors.

## 1. Introduction

Poultry meat is an inexpensive and good source of animal protein. Poultry meat generally originates from broiler meat such as chicken, turkey, quail, and guinea fowl meat. Poultry breeding usually focuses on the live weight and eggs of birds [1]. In the agricultural sector, poultry farming plays an important role in economic growth, market supply, and farmers’ income. However, the emergence of multidrug resistance (MDR) poses a serious challenge to global health, severely compromising the efficacy of antibiotics in treating bacterial infections [2,3,4]. The growing threat of antimicrobial resistance in disease-causing bacteria, particularly those affecting farm animals, such as poultry, has become a critical worldwide concern. The World Health Organization (WHO) ranks antimicrobial resistance (AMR) among the top 10 threats to global public health [5]. Its repercussions include diminished antibiotic effectiveness; extended periods of sickness; increased death rates; and sustained financial losses across various industries, such as farming and food manufacturing [6].

Excessive and inappropriate use of antibiotics in the poultry industry has led to the rapid development of antibiotic-resistant bacteria. Among the most prevalent pathogens affecting the health of poultry are *Escherichia coli* (*E. coli*), *Salmonella* spp., *Staphylococcus aureus*, and *Enterococcus* spp., which have become increasingly resistant to antibiotics owing to their widespread and often improper application in poultry farming [7]. Since poultry plays an essential role in the global food supply, especially in developing countries, it is vital to find new, more effective, and safer sources of growth promoters [8]. Antimicrobial agents found in the essential oils of herbs and spices have been shown to have the potential for use as alternatives based on their inherent properties [9].

**Figure 1:**
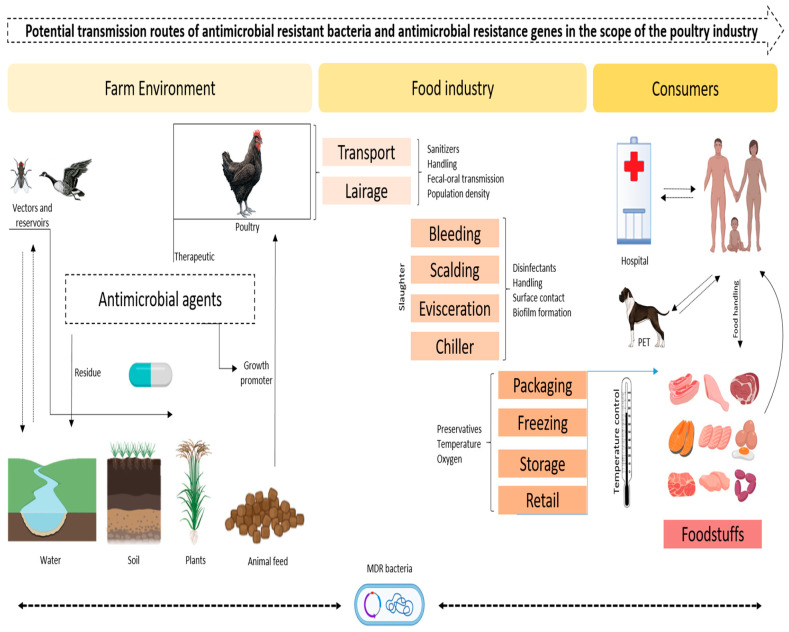
Possible pathways of antimicrobial-resistant bacteria and A/M resistance genes in the context of the poultry industry (designed with Biorender.com).

Figure 1 showing, The poultry industry is one of the important industries through which ARB (antimicrobial-resistant bacteria) and ARGs (antimicrobial resistance genes) can easily transfer from one point to another through various associated pathways that are potentially hazardous to both animals and humans. The identified transmission routes include direct contact between infected poultry and farm workers; the consumption of contaminated poultry meat or products; and, lastly, the environment through cropping with the use of manure, litter and wastewater. Antibiotics are used on poultry farms for growth promotion or disease prophylaxis, and such practices promote resistance in bacteria. Specifically, resistant strains and their related genes can be transmitted to humans through contact with poultry and its products or the polluted environments of soil and water supplies. The movement of PPE (Personal Protective Equipment) also enhances the dissemination of ARB and ARGs across regions, thereby compounding the public health issue of AMR (antimicrobial resistance).

AMR in poultry represents a major global health concern. Antibiotics are still widely used in poultry production for growth promotion, disease prevention, and treatment, creating conditions that favor the evolution of resistant bacteria. This review was therefore conducted to address existing knowledge gaps and synthesize current evidence. However, despite substantial evidence establishing a link between poultry farming and AMR, there is a lack of understanding of the mechanisms and transmission pathways of resistant bacteria, particularly from animals to humans and the environment. Key research issues include the identification of alternative growth promoters, AMR dynamic transmission, and integrated management strategies under the One Health framework [10,11,12]. AMR in poultry production systems is a risk that should be addressed by targeted research, cross-sectoral collaboration, and policy reforms [13]. This review discusses the main reasons for AMR in poultry and the contribution of alternative strategies, such as probiotics and vaccines, as well as presenting recommendations for more complete surveillance and regulatory frameworks to control AMR.

## 2. Study Selection Criteria

**Figure 2:**
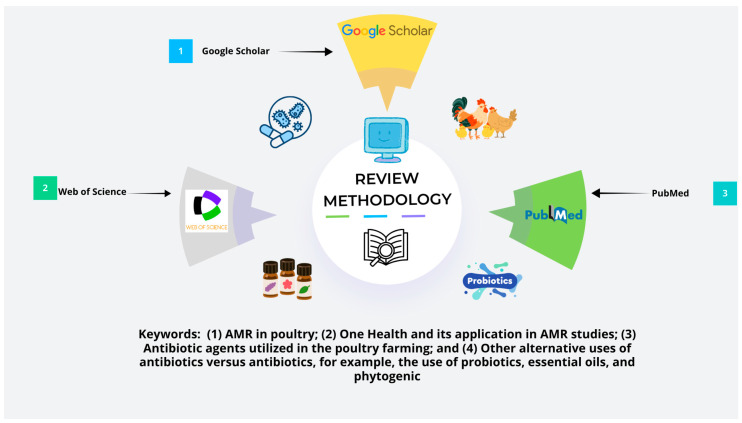
Review methodology used for selection of keywords from online databases like Google Scholar, Web of Science, and PubMed.

A number of relevant studies (a total of 198 studies were found, among which 123 were closely related to the relevant keywords searched) were found in various databases such as PubMed, Scopus, Web of Science, and Google Scholar. For example, the included studies were related to (1) AMR in poultry; (2) One Health and its application in AMR studies; (3) the antibiotic agents utilized in poultry farming; and (4) the use of alternative antibiotic agents versus antibiotics, such as the use of probiotics, essential oils, and phytogenic agents. Studies that were not directly linked to poultry farming or AMR, that were not written in the English language, or that were purely based on opinions without any supporting empirical data were excluded. A total of 108 studies were included, and the newly developed tool Consensus helped to find relevant research articles using artificial intelligence. What is Consensus? It is an online website (search engine) designed to help researchers find relevant studies using relevant keywords and questions in an online database (for more details, visit www.consensus.app). First, information was compiled from various studies from different locations to determine the challenges in AMR development and AMR in poultry production patterns. Second, we aimed to determine how the One Health framework can be applied to solve AMR. Similarly, this approach was also used to ensure a complete review of the current knowledge on AMR in poultry, the efficacy of other strategies, and policy changes, as shown in Figure 2 below.

## 3. Antibiotic Resistance and Its Health Perspective

Antimicrobial resistance (AMR) is a critical global health problem that affects animals, humans, and the environment interdependently [14,15,16,17,18]. The “One Health” concern is directly connected to the improper usage of antibiotics in industry, which creates different types of pressures on resistant bacterial strains [19]. The main causes of this problem are medication management and administration systems. Thus, pharmaceutical management organizations need to protect patients or the supply; however, this issue is spreading and becoming an important multidrug-resistant problem [20]. From a single health perspective, antimicrobial resistance is interconnected with nature and presents risks and threats to human and animal health. The “One Health Perspective” supports the use of antibiotics for the environment, human health, and animal health. Thus, taking care of animals, humans, and nature is the prime motive of the “One Health” approach [21].

**Figure 3:**
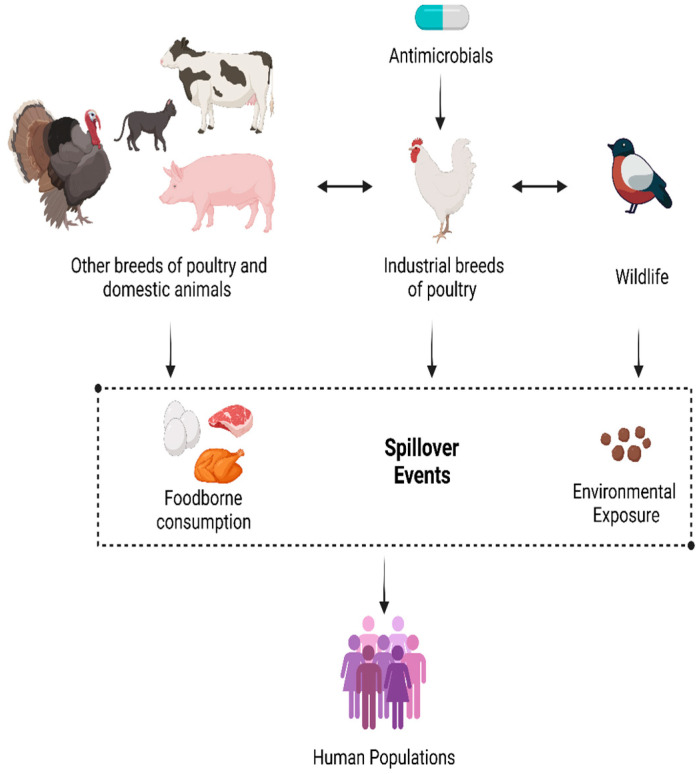
Antimicrobial resistance in poultry farming within low-resource settings (designed with Biorender.com).

Figure 3 showing below, Antimicrobial resistance (AMR) in poultry farming within low-resource settings is a growing concern, driven by factors ranging from limited access to veterinary oversight, poor biosecurity measures, and unregulated antibiotic use. In this environment, antibiotics are applied for growth promotion, disease prophylaxis, and curative purposes, increasing the risk of further antibacterial resistant-bacterial strains. Due to poor waste disposal methods, AMR and its causes—ARB and ARGs—are transferred through the soil, water, and air. Furthermore, the lack of an effective monitoring and supervision system allows AMR to grow uncontrollably, which may pose broader threats to regional and global health. In low-resource settings, reducing the threat of AMR necessitates the immediate adoption of sustainable agriculture, promotion of the responsible use of antibiotics, and effective infrastructure to address biosecurity and waste management.

## 4. Use of Antibiotics in Poultry

Among these, tetracyclines, fluoroquinolones, macrolides, sulfonamides, aminoglycosides, polypeptides, and a group of beta-lactam antibiotics have been identified in poultry [22,23,24,25]. Government investigations have linked the use of antibiotics in poultry and livestock farming to multidrug resistance, a factor that is dangerous to both animals and humans. Bacteria originating from animals are capable of infecting and colonizing humans, highlighting the relationship between antibiotic usage in food animals and antibiotic resistance.

## 5. Tetracyclines

Tetracyclines are antibacterial agents that exhibit activity against both Gram-positive and Gram-negative bacteria. They are often used to treat respiratory and mycoplasma infections, with the latter being rife in poultry. Tetracycline resistance genes are often reported in the environment, thereby enhancing the spread of resistance [26,27].

## 6. Fluoroquinolones

Enrofloxacin and ciprofloxacin are used to treat respiratory tract and enteric bacterial infections that commonly affect farm animals. In poultry, they are the most effective against *Escherichia coli* and *Salmonella* spp., and their misuse has led to the development of resistance [28].

## 7. Sulfonamides

The most famous sulfonamides are sulfamethoxazole and sulfadiazine, which are used in conjunction with trimethoprim. These combinations have been administered against *Escherichia coli* and coccidiosis in poultry. Susceptibility to sulfonamides remains a problem in animals raised for food production [29].

## 8. Aminoglycosides

Some examples of feed antibiotics include aminoglycosides of gentamycin and neomycin, which are used for the treatment of severe bacterial diseases in poultry production. They are most active against *Escherichia coli* and *Salmonella* spp. but are resistant to aminoglycosides; thus, their use remains somewhat restricted [30,31].

## 9. Polypeptides

Polypeptides, including colistin, are used as narrow-spectrum antibiotics to control bacterial infections, such as *Escherichia coli* and *Salmonella* spp., in poultry. As a last resort for certain infections, colistin is extensively used in animal feed; thus, it is partly responsible for the development of resistance [28].

## 10. Beta-Lactams

Different groups of beta-lactam antibiotics, such as penicillin (amoxicillin) and cephalosporin, are used in the management of different bacterial infections in poultry. These antibiotics are used to treat respiratory infections caused by *Escherichia coli* and *Pasteurella multocida* and necrotic enteritis caused by *Clostridium perfringens*. Cephalosporins are mainly used to treat *Escherichia coli* (or *colibacillosis*) and *Salmonella* spp. However, for this reason, they are associated with the development of extended-spectrum beta-lactamase (ESBL)-producing bacteria, leading to lower effectiveness of other beta-lactams in animals and humans [32,33].

## 11. Bacterial Vaccines

Bacterial vaccines are key approaches that help decrease the impact of AMR in poultry, since the use of antibiotics for disease prevention and control is limited. These vaccines help activate the immune system of poultry to fight certain bacterial diseases affecting poultry and are associated with antimicrobial-resistant infections, including *Salmonella* spp. and *Escherichia coli*, in poultry production. However, some constraints, including the cost of developing expensive vaccines, the availability of these vaccines to small-scale farming communities, and the appropriateness of vaccine administration, still present challenges that need to be overcome. Therefore, the application of bacterial vaccines as part of integrated AMR control programs is a sustainable strategy for maintaining antibiotic effectiveness and population health.

**Figure 4:**
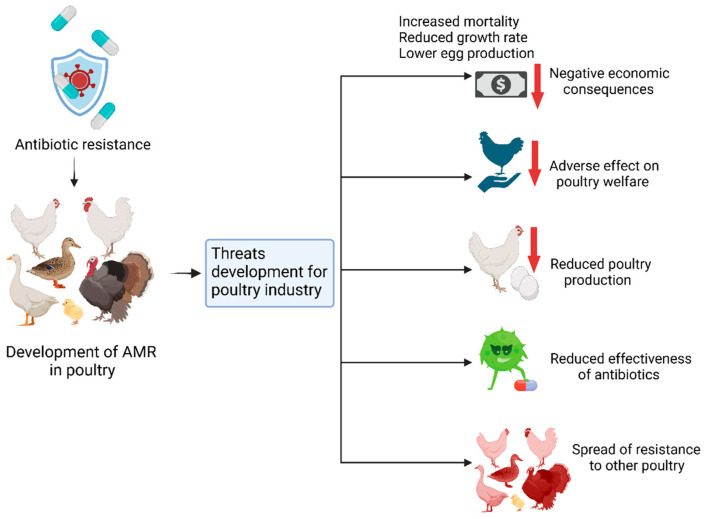
Bacterial vaccines combating antimicrobial resistance in poultry (source: created using Biorender.com).

Figure 4 showing below Bacterial vaccination has been identified as an essential preventive measure to eliminate the use of antibiotics and prevent AMR in poultry farming. Since vaccines protect poultry against specific bacterial pathogens like *Salmonella*, *Escherichia coli*, and *Pasteurella multocida*, they prevent diseases that would otherwise need antibiotics. Thus, this approach reduces selective pressures on bacterial populations that are responsible for the development and persistence of resistant forms. Vaccination not only improves the health and egg productivity of poultry flocks but also provides a rare opportunity to minimize the circulation of antimicrobial-resistant bacteria in processed poultry products and, thus, the exposure of consumers to resistant pathogens. Further, bacterial vaccination enhances the production of sustainable livestock by providing a cure for bacterial diseases, thus avoiding the misuse and overuse of antibiotics in animal feed as growth promoters or remedies for diseases. For optimum results, bacterial vaccination programs should be accompanied by enhanced biosecurity measures, farmer profiling, and effective monitoring programs. This post-AMR inclusive approach can potentially reduce AMR considerably and, at the same time, improve animal health and the sustained effectiveness of the antibiotics.

Table 1 provides a clear description of the six groups of antibiotics widely used in poultry farming, how they are used, examples of antibiotics in each group, and their resistance challenges. Among them, tetracyclines, fluoroquinolones, sulfonamides, aminoglycosides, polypeptides, and beta-lactams are used to treat bacterial infections, respiratory diseases, *Escherichia coli*, and *Salmonella* spp. However, the abuse of these antibiotics has caused major resistance hurdles, such as the dispersal of resistance genes in the environment, decreased drug effectiveness, and the emergence of extended-spectrum β-lactamase (ESBL)-producing bacteria.

## 12. Antibiotic Resistance in Poultry

Antibiotic resistance in poultry refers to the ability of bacteria to evade the effects of antimicrobial agents intended to inhibit or eliminate them [34,35,36]. These mechanisms of resistance are dangerous to the health of both the animals themselves and humans, given that the genes offering resistance make their way into different populations. The main sources of resistance in poultry include enzymes, target receptors, efflux systems, gene transfer, biofilm formation, changes in membrane permeability, and plasmid transfer.

## 13. Enzyme Degradation

Cheminformatics shows that bacteria use enzyme degradation as one of the main resistance strategies, where bacteria produce enzymes that can modify or degrade antibiotics. For example, there is a phenomenon where resistant bacteria can inactivate antibodies by hydrolysis or some other action related to structural changes. These include beta-lactamases and aminoglycosides. Extended-spectrum beta-lactamases alter the penicillanic acid moiety and the 7 alpha–methyl group of cephalosporins, making them unable to bind to suitable targets on bacteria. Studies reported that this mechanism greatly compromised the effectiveness of these important antibiotics [26,27,28].

## 14. Target Site Alterations

Another form of resistance can also occur through changes in the structure of the target placed on the bacteria to which the antibiotic is supposed to bind. This mechanism is commonly identified in fluoroquinolone-resistant *Campylobacter jejuni* and *Escherichia coli* in poultry. For example, alteration in the gyrA locus, which codes for DNA gyrase, is the basis of fluoroquinolone resistance in *Campylobacter jejuni* and *Salmonella* spp. Likewise, methylation of 23S ribosomal RNA by erm genes leads to macrolide resistance, as *Campylobacter jejuni* strains are resistant to erythromycin [37].

## 15. Efflux Pumps

Efflux pumps are membrane proteins that pump out antibiotics from prokaryotic cells, thereby decreasing the intracellular accumulation of the drug to non-lethal levels. A major function of these pumps is to expel numerous types of antibiotics, including tetracyclines, macrolides, fluoroquinolones, and many others, that accumulate in bacteria, leading to multidrug resistance. Some known examples are Tet (A)and Tet (K) efflux proteins in tetracycline-resistant *Escherichia coli* and CmeABC in *Campylobacter jejuni*, which are responsible for macrolide and tetracycline resistance [38,39,40].

## 16. Horizontal Gene Transfer

Horizontal gene transfer refers to the process by which exogenous resistance genes are inserted into the recipient bacterium from another bacterium through plasmids, bacteriophages, and other mobile genetic elements. Such a transformation can occur through transduction, which is the process whereby bacteriophages can cause the transfer of resistance genes between bacteria. For instance, the mcr-1 gene, which confers resistance to colistin, has been identified in *Escherichia coli* and *Salmonella* spp. in poultry-producing farms. A high population density means that birds live in conditions that can easily spread resistant bacteria from one farm to another [41,42].

## 17. Biofilm Formation

Biofilms are bacterial surface layers that offer protection from antimicrobial agents and disinfectants. Since bacteria grow in biofilms during poultry production, the resulting infections with *Salmonella* spp. and *Campylobacter jejuni* become more difficult to eradicate and manage [43,44,45].

## 18. Reduced Membrane Permeability

Certain bacteria decrease their membrane fluidity by downregulating porins, which are proteins that create aqueous pores through the bacterial outer membrane. These changes have hindered the accessibility of most antibiotics with consequential resistance. For instance, in *E. coli*, resistance to beta-lactams and carbapenems is associated with low permeability in Gram-negative bacteria [46,47].

## 19. Plasmid-Mediated Resistance

Plasmids are currently recognized as important vehicles for the dissemination of resistance genes, such as colistin (mcr-1) and extended-spectrum beta-lactamases (ESBLs), to inactivate beta-lactam antibiotics. This mechanism is widely used in poultry owing to the widespread use of antibiotics in growth promotion and disease prevention. In this context, the ability of plasmids to move from one cell to another allows the rapid dissemination of resistance both within a species and between them, thus making the problem of antibiotic resistance control even more difficult [48,49].

## 20. Effects of Antibiotic Resistance on Human Lives

AMR in poultry production has many implications for human health. It reduces the effectiveness of medical interventions, raises mortality, causes various economic losses, and spreads zoonotic-resistant pathogens [50,51]. Furthermore, pollution and gene swapping make AMR a threat to public health and ecosystems.

## 21. Treatment Failures

Resistance to antibiotics reduces the efficacy of the treatments applied, and the length of disease, complication rate, and treatment outcome are all adversely affected. The conjugation of foodborne pathogens, such as Salmonella spp. and Campylobacter from poultry, makes infection even more challenging to treat. Second, an increasing number of multidrug-resistant bacteria such as K. pneumoniae and Escherichia coli have pushed carbapenems and colistin, the last-resort antibiotics, to cautionary use, as they restrict drug effectiveness and impair treatment results [52,53].

## 22. Increased Mortality

Antibiotic treatment failure due to antibiotic resistance is associated with increased mortality rates. For instance, MRSA infections cause bloodstream infections that are deadly and result in high mortality rates. Similarly, carbapenem-resistant Enterobacteriaceae are responsible for invasive infections, with mortality rates ranging from 30 to 50%. If no action is taken, antibiotic resistance may cause 10 million deaths a year by 2050, which defines it as a severe threat to world health [53,54,55].

## 23. Economic Burden

Antibiotic resistance drastically affects every healthcare system worldwide and is associated with heavy economic costs. Clinicians dealing with patients with resistant infections are likely to spend more time in the hospital, request more tests, and use more expensive treatments. The combined impact of these factors is estimated to lead to a global economic loss of USD 100 Tn by 2050, making it crucial to address AMR [53,54,55,56,57].

## 24. Zoonotic Disease as a Public Health Threat

The cross-species transmission of resistant bacteria from poultry to humans represents a severe threat to public health. The handling of animals in production and processing enterprises, such as by farm workers, can lead to direct contact with organisms such as *Escherichia coli* and *Campylobacter jejuni*. Moreover, eating dead poultry products such as bird meat, dairy, and eggs escalates the risk of transferring more dangerous strains, such as *Salmonella* spp. and *Campylobacter jejuni,* to humans as a result of undercooking [57,58,59,60,61].

## 25. Environmental Routes

Poultry farming exposes bacteria to antibiotics, leading to resistance in water, soil, and crops from discarded waste. For example, *Escherichia coli* and other *Enterobacteriaceae* found in poultry waste can contaminate water, causing unknown indirect routes to humans. This type of environmental pollution worsens the public health implications of AMR [59,60,62].

## 26. Horizontal Gene Transfer

Resistance genes from animal-associated bacteria can be transferred to human pathogens through horizontal gene transfer mechanisms, including plasmids and other mobile genetic elements. This transfer increases the diffusion rate of resistance with genes found in plasmids, such as the mcr-1 gene, which contributes to resistance to antibiotics as crucial as colistin. In crowded poultry farming conditions, resistance genes add an extra impetus for the transfer of infections prone to resistance in humans [46,47,48,49,63,64].

The biological basis, examples of resistant bacteria, the impact on poultry and human health, and possible strategies for the prevention of resistance mechanisms in poultry are presented in Table 2. Additionally, it describes the difficulties encountered in countering each of these resistance mechanisms and calls for integrative, farm-level biosecurity and antibiotic stewardship at the farm and global levels.

## 27. Challenges in Addressing Antibiotic Resistance in Poultry

The significance of antimicrobial resistance (AMR) in poultry farming is an urgent issue affecting human and animal health [13,65,66,67]. The widespread and often inappropriate use of antibiotics in agricultural practices has contributed significantly to the rise of AMR; many farmers use antibiotics intensively and in an uncontrolled manner. There are several critical issues that need to be addressed to minimize this issue, including better antibiotic stewardship; a decrease in unnecessary use, such as in farming; and alternative solutions such as essential oil to prevent the development of resistance.

## 28. Appropriate and Casual Use of Antibiotics

Inappropriate use of antibiotics by individuals and businesses is one of the main causes of AMR in poultry production [68]. However, these medications are frequently used to prevent illnesses and as feed additives to increase productivity in less-developed countries. Thus, this strategy promotes the selection and dissemination of resistant bacterial strains throughout the environment more quickly. Furthermore, there are no low-cost, effective alternatives that delicately address this issue, such as probiotics, prebiotics, and vaccines. Because of their high cost and the practical difficulties in implementing them, it has become clear that these options are either unavailable or rarely used in many places.

## 29. Economic Pressures

The improper use of antibiotics in poultry is mainly attributed to economic factors that poultry farmers face, especially in L & MICs [69,70]. Agriculturalists are operating on very tight profit margins and are eager to achieve optimum yields; thus, they feed their animals with antibiotics not only for growth purposes but also for disease control. Moving to organic farming or using antibiotics can be costly, making switching impossible for most farmers.

## 30. Awareness Gaps

The proper use of antibiotics and the effects of antibiotic resistance are not well understood by smallholder poultry producers. However, producers are also not very motivated to practice responsibly because of the lack of public awareness and demand for poultry free of antibiotics. Campaigns for consumer and farmer education is necessary to address these disparities.

## 31. Monitoring and Surveillance

The screening and control of antibiotic use and resistance are crucial but generally inadequate. The lack of adequate capital and equipment in most areas prevents extensive data collection and analysis. This also makes antibiotic resistance a global problem, because such bacteria may spread through international trade and, for instance, migratory birds. This reveals the necessity for harmonized and planned worldwide observation systems.

## 32. Pathogen Evolution

Previous work has suggested that pathogens within poultry are capable of developing resistance to antibiotics quickly because of selective pressure. Another way that antibiotic residues contribute to this problem is through poultry waste, which becomes a new environmental reservoir for these genes. Several of these genes are mobile and can move from one species of bacteria to another, creating a more formidable and dangerous setup for humans and animals.

## 33. Policy and Multistakeholder Management

AMR prevention strategies in the poultry production chain are usually poorly integrated among the human, veterinary, and agricultural sectors [10]. The biggest challenge is achieving effective coordination under a One Health framework, as these sectors are interrelated. However, these global endeavors face many challenges, such as differences in funding and emphasis between developed and developing countries [11]. In essence, the attainment of robust and effective global strategies entails huge capital investments, political determination, and multilateralism. Therefore, it is necessary to conclude that the problem of antibiotic resistance in poultry production is complex, and it can only be solved through the adoption and implementation of legal norms, the provision of financial incentives, adaptive monitoring, and international cooperation. Intervention and prevention from all stakeholders are necessary to fight the implications of AMR by applying the One Health concept [12]. Implementation efforts for the responsible use of antibiotics depend on the coordination of multiple sectors, including human, animal, and environmental coordination, which involves cross-sectoral cooperation. Examples of successful collaboration are specific, such as the creation of transdisciplinary networks like the UK-based initiative, which seeks to link farm-to-fork stakeholders and harmonize AMR-related activities across the agriculture, healthcare, and public health sectors [71]. Likewise, in Vietnam, a One Health approach to AMR surveillance has led to the strengthening of multisector collaboration between the animal and human health sectors, awareness of the importance of sharing data, and the harmonization of policy frameworks [72]. In addition, the need for cross-sectional coordination of the national AMR surveillance system was assessed with recommendations for the establishment of a central entity with a mandate to develop and maintain a framework at the national level for AMR surveillance and data sharing among human, animal, and environmental health surveillance programs [73]. It is significant to say that the importance of cross-departmental collaboration to achieve AMR management, which consists of a link between policies and consolidated surveillance networks, cannot be overemphasized.

In 2006, the European Union (EU) implemented a radical policy change by banning the use of antibiotics as growth promoters in livestock in response to the antibiotic ban initiative, as the associated AMR posed a threat to public health [11]. Research has revealed that the feeding of antibiotics to animals to promote growth leads to the spread of resistant bacteria, which also pass to humans directly or through the food chain; therefore, there is a need to ban the use of these antibiotics [13,65,66,67]. Nevertheless, this procedure is used in other countries worldwide, which, along with others, is the main cause of the global AMR crisis, and many countries, mainly in Europe, have forbidden its use for growth promotion since the ban. There is a lack of reviews discussing these legal frameworks and their effects on the global use of antibiotics. Moreover, the bans have resulted in the modification of farming practices and have led to the exploration of new methods for controlling animal health as a way of stamping out AMR.

## 34. Alternatives to Antibiotics

There are alternative means of using or not using antibiotics, such as avoiding or reducing their use to prevent and treat infections or to promote the health and growth of animals and humans. The purpose of this study was to solve the global antimicrobial resistance (AMR) problem without adding to the spread of resistant pathogens while maintaining efficacy in disease control. The main characteristics are a non-antibiotic mode of action to kill microbial infection, promotion of host immunity or gut health, targeting of pathogens without harming the beneficial microbiota, and reducing dependency on conventional antibiotics, especially in agriculture and food systems. The rise in AMR has prompted growing interest in exploring alternatives to antibiotics in poultry. Such alternatives help maintain animal health and productivity while minimizing the risks associated with AMR. Non-antibiotics include natural, synthetic, or biological products that can either target pathogens or support host defenses. Alternatives to antibiotics include actions to control infectious diseases and promote animal health without the use of antimicrobial agents. These substitutes are used for antimicrobial resistance by providing mechanisms of pathogen suppression or immune modulation independent of antibiotic pathways. Some alternatives are described below.

## 35. Probiotics

Probiotics are involved in the improvement of gut health and immunity in poultry. Probiotics are living microorganisms that colonize gut epithelial surfaces; produce antimicrobial compounds, such as bacteriocins and organic acids; and stimulate the immune system through gut-associated lymphoid tissue (GALT) [74,75,76]. They also reduce the levels of *Salmonella* spp. and *Escherichia coli* and improve feed efficiency and growth rates [77,78,79]. Challenges such as strain-specific effects, variability in efficacy, and stability during storage and feed processing may occur. Examples include *Lactobacillus*, *Bifidobacterium*, and *S. boulardii.*

## 36. Prebiotics

Prebiotics are indigestible food ingredients that promote the growth of gut bacteria. They serve as substrates for important microbes and enhance the production of short-chain fatty acids (SCFAS). They lower the pH of the gut to restrain or prevent the growth of pathogenic bacteria. Prebiotics also improve the gut microbiota and enhance nutrient absorption. The challenges that are mainly faced when using prebiotics are that they have higher costs while being applicable [80]. Examples include mannan oligosaccharides, fructooligosaccharides, and inulin.

## 37. Phytogenics

Phytogenics, also known as phytobiotics or botanicals, are plant-based compounds used in animal nutrition to promote growth, improve feed efficiency, and enhance overall health [81]. These include a wide variety of bioactive substances such as essential oils, flavonoids, alkaloids, tannins, and saponins. They disrupt bacterial cell membranes, produce toxins, and exhibit anti-inflammatory and antioxidant properties. They have broad-spectrum antimicrobial effects, and they improve feed palatability and digestion. They present challenges in terms of variability in composition and efficacy, owing to their natural sources.

## 38. Enzymes

Enzymes play a role in improving nutrient digestion, enhancing feed digestibility, and reducing undigested material in the gut [82]. They break down or reduce nutrient factors in the feed of pathogenic bacteria. They improve the absorption of nutrients and feed efficacy and reduce intestinal stress, and the main challenges of stability arise during feed processing. Poultry feed ingredients, such as cereals and legumes, contain anti-nutritional factors, such as non-starch polysaccharides, phytic acid, and protease inhibitors, which absorb nutrients. Phytic acid is an important enzyme in poultry feed that enhances nutrients by breaking down phytate, and it produces an indigestible form of phosphorus in plant-based feed. Phytate is poorly digested by poultry, owing to the absence of phytase in the gastrointestinal tract. Phytic acid releases phosphorus, calcium, and minerals, and it improves bone health in poultry, lowering feed costs and environmental phosphorus pollution. Xylanase hydrolyzes xylan [82].

The breakdown of non-starch polysaccharides, which are found in the hemicellulose of plant cell walls, such as binoxylans and cellulose, is observed in cereals such as wheat barley and rye. Xylan in wheat, rye, and barley increases intestinal viscosity; reduces nutrient absorption; reduces viscosity; enhances digestion; and frees nutrients trapped within plant cell walls, such as starch, proteins, and lipids. Its role in poultry nutrition is to improve the feed conversion ratio (FCR) [83,84,85,86]. It is primarily used in wheat- and barley-based diets to reduce wet litter and to improve gut health. This enzyme hydrolyzes cellulose, a major component of plant cell walls, into glucose and other smaller carbohydrates [87]. Breaking down of fiber means converting indigestible cellulose into absorbable sugars, thus providing an energy source and improving gut motility and nutrient utilization. It also enhances nutrients such as proteins and starch within plant cells. Protease is an effective enzyme that is commonly used in poultry production. Protease enzymes target the proteins in feed to cut or break down small peptides and amino acids that are easily absorbed. Proteases help improve digestibility, including that of protein feed ingredients, and feed efficiency, and they support gut health. A group of enzymes hydrolyze the peptide bonds in proteins. Endogenous proteins such as pepsin and trypsin are insufficient to fully digest the plant-based and protein-rich ingredients in poultry feed [88]. They increase bioavailability and neutralize protein-based anti-nutritional factors such as trypsin inhibitors found in legumes.

The digestibility of proteins ensures maximum nutrient absorption, thereby reducing the need for high-protein diets. It enhances protein digestion, minimizes undigested protein entering the lower gut, and reduces nitrogen excretion in the feces. This is crucial to environmental sustainability. Proteases improve the efficiency of feed utilization, which reduces feed costs while maintaining and improving production. Trypsin inhibitors in legumes interfere with protein digestion, and their supplementation breaks them down owing to their enhanced feed efficacy. Undigested proteins in the gut provide a surface for the growth of pathogenic bacteria such as *Clostridium perfringens*. Proteases reduce the given surface by supporting gut health and reducing the risk of diseases such as necrotic enteritis.

## 39. Essential Oils in Poultry

Anti-cracking, anti-inflammatory, antioxidant, and growth-promoting essential oils can be extracted from plants. These topical essential oils offer promise as alternatives to antibiotics and promote gut health and production without increasing antibiotic resistance [89]. Essential oils are mixtures of volatile compounds, mostly terpenes; phenolics; alcohols; and bioactive components, such as thymol, carvacrol, cinnamaldehyde, and eugenol. Essential oils are also antibacterial, as they disturb bacterial cell membranes and prevent bacterial activities, such as enzyme activity and DNA synthesis. They are effective against a wide variety of pathogenic bacteria, including *Campylobacter jejuni, Escherichia coli*, and *Clostridium perfringens*.

Pathogenic bacteria or stress can lead to allergic inflammation in the intestine, leading to a decrease in essential oil levels [90]. The feed conversion ratio (FCR) and growth rate improve with the use of essential oils, as they ensure the health of the gut and the germination of the nutrients. Birds fed supplementary diet additives such as oregano oil are more efficient in terms of weight and feed. The antimicrobial effects of oregano oil reduce the pathogen load in the gastrointestinal tract of poultry, facilitate digestion and nutrient absorption, and ultimately improve overall poultry growth performance.

## 40. Essential Oils with Active Compounds and their Applications

The use of antibiotics has decreased, and interest in natural alternatives to antibiotic agents has increased; therefore, the current study analyzes the effects of essential oils in poultry production. The bioactive compounds in these oils support the gut health of animals, increase productivity and antimicrobial activity, and are important tools in sustainable agriculture.

## 41. Oregano Oil (*Origanum vulgare*)

Carvacrol and thymol are active compounds present in oregano oil. These components show powerful antibacterial activity against pathogens, such as *Escherichia coli* and *Salmonella* spp. Moreover, oregano oil enhances the FCR and body weight gain in poultry. This, in turn, over and above supporting a balanced gut flora, makes the substrate useful in maintaining a bird’s general health and productivity [91].

## 42. Thyme Oil (*Thymus vulgaris*)

The major constituents of thyme oil include thymol and carvacrol, which make it very useful [92,93]. It increases the level of enzymatic activity of digestive enzymes involved in enhanced nutrient assimilation. To summarize the applications of thyme oil, it is a potent source of antioxidants and antimicrobial agents, minimizes intestinal lesions due to pathogenic organisms, and, as such, makes for a great supplement to feed additives for a healthy gut and disease tolerance [94].

## 43. Peppermint Oil (*Mentha piperita*)

The main active ingredients of peppermint oil are menthol and menthone. In addition to enhancing feed palatability, this essential oil can also help decrease heat stress in poultry, which is very important for improving the production performance of poultry under hot conditions. In addition, peppermint oil is beneficial for the health of birds because it improves their gut health [95].

## 44. Rosemary Oil (*Rosmarinus officinalis*)

Rosemary oil contains large amounts of rosmarinic acid and carnosic acid. Rich in antioxidant properties, rosemary oil is used to improve the quality of meat and increase its shelf life. This also aids gut health, promoting overall health and performance in poultry production [96]. Therefore, adding the above important oils to poultry feed or water could be a novel strategy if applied in place of antibiotics to enhance production standards and bird health.

Despite the discussion of the potential of alternatives to antibiotics, such as probiotics, essential oils, and phytogenic agents, most of the available research is theoretical and lacks field trials to prove whether their use in commercial poultry production is effective. Lactobacillus and Bifidobacterium have been found to improve gut health and reduce *Salmonella* spp. and *Escherichia coli* pathogens [97], whereas essential oils such as oregano and thyme have antimicrobial and anti-inflammatory properties that may promote poultry health without promoting antimicrobial resistance [98]. However, such studies are conducted in controlled lab environments and at small scales, thus providing an incomplete understanding of the effectiveness of these tools under different field conditions. A number of these alternatives should be tested more robustly under large-scale trial conditions to determine how they perform in different geographical locations, farming practices, and poultry species.

In addition, cost–benefit analyses are often missing from such discussions, even though they are crucial in determining the feasibility of the widespread adoption of such alternatives. Studies have shown that these non-antibiotic additives have a positive effect on growth performance and feed efficiency; however, their economic viability is unclear. Probiotic and essential oil-based alternatives are often much more expensive than traditional antibiotics, which sparks doubt over their applicability in certain types of farms, such as those run at small scales or with thin profit margins. These alternatives must be assessed at the project level to determine whether they are cost-effective compared with alternatives and whether they can be integrated into commercial poultry systems without causing financial strain [99].

## 45. One Health Framework: A Deeper Analysis of Antimicrobial Resistance Transmission Mechanisms

Antimicrobial resistance (AMR) is a complex issue that cannot be addressed without referring to the “One Health” concept, that is, the idea that the health of humans, animals, and the environment is interrelated. However, the current literature on the dynamics by which antimicrobial resistance genes (ARGs) spread across these connected domains remains insufficient. Although this article discusses AMR as a One Health issue, it does not delve into the migration pathways through which resistant bacteria and ARGs move among humans, animals, and the environment. There is evidence that environmental contamination, as in water bodies or soil, is a critical driver of the dissemination of ARGs, which are then transferred between species through ecological interactions [100,101]. To completely understand the core of AMR transmission, these pathways are fundamental to realizing that environmental reservoirs, including wastewater, agricultural runoff, and contaminated surfaces, are a robust source of resistance gene flow to all biological and ecological systems.

Specifically, it has been demonstrated that resistant bacteria spread through the global environment from waste generated by animal farming, human healthcare settings, and environmental pollution [102]. For instance, AMR *Escherichia coli* strains are found in both urban and rural lakes and are resistant to both aquatic and non-clinical ecosystems, indicating that non-clinical ecosystems can contain large reservoirs of AMR strains that can threaten human and animal populations [103]. Furthermore, the mobility of mobile genetic elements (MGEs), such as plasmids with resistance genes, enhances the dissemination of AMR among environmental sources and hosts. Current knowledge on AMR fails to address the critical transmission channels, and interventions that cover the continuum of the human–animal environment are hampered. Monitoring and mitigating the risk of AMR spread should encompass a comprehensive One Health approach, including all ecological and biological systems, as it permits a more complete evaluation of the role of environmental media and cross-species transmission in AMR persistence and spread [71,104].

## 46. Antibiotic Use and Resistance in Poultry Farming in Developing Countries

Antimicrobial use in poultry farming and other sectors is widespread in developing countries, where it may not be well controlled and is among the several factors contributing to the spread of antimicrobial resistance (AMR). In Pakistan, poultry farms were given no-prescription antibiotics, such as amoxicillin and colistin, which only added fuel to the fire of the problem of resistance [105]. Similarly, farmers in South Asia, including Bangladesh, India, and Nepal, use antibiotics that are routinely used to promote growth and prevent diseases without supervision. In addition, this practice promotes resistance in poultry, increasing the chance that resistant bacteria are transmitted to humans through the food chain [106]. Surveillance does not work, and there is an inclination to use tetracyclines and fluoroquinolone drugs indiscriminately, which contributes to the issue, making it a vital open health concern in the area [107]. This suggests the need for more effective regulations, education, and surveillance to stop the rapid rise of AMR in developing countries.

## 47. Future Directions

As the use of antibiotics escalates to fight resistant bacteria in poultry, a complex approach awaits research, policy, public health education, and global collaboration, known as One Health. Plans should aim to further develop alternative approaches, including probiotics, prebiotics, phytogenic agents, and enzymes; sensibly reduce the cost of these feed additives; and enhance their availability, especially in LMICs. Prolonged funding for advanced technologies, such as microbiome modulation and precision agriculture, has the potential to improve poultry health, but not through antibiotics. It is important to further enhance the global surveillance of antibiotic use and resistance. This encompasses synchronizing methods of obtaining data and encouraging cooperation in monitoring at the international level, as well as containing the spread of resistant bacteria. Antimicrobial-free production needs to be incentivized, and substantial funds need to be allocated to support farmers in changing from conventional methods. Public awareness initiatives for producers and consumers are required to promote sound judgment and timely demand for products that are free from antibiotics. It is important to reduce awareness gaps and conduct reviews involving resistance by training communities in poultry farming, health promotion, and other campaigns, including reminding farmers of the appropriate practices. There is also a need to enhance existing legal instruments to provide better legal support for the restrictions on antibiotic utilization while encouraging the proper utilization of more prolonged sustainable substitutes. These global initiatives must be able to balance the differences between developed and developing countries in terms of the availability of resources and knowledge to tackle AMR.

Antibiotic resistance in poultry increases the cost/kg by reducing the feed conversion ratio; prolonging the time to market; and increasing mortality, treatment, and downtime costs at the farm level. At the processor and market levels, AMR findings result in carcass condemnation, store delisting, and export rejections, while residue compliance and monitoring initiatives incur ongoing expenditures and impede throughput. From a One Health perspective, risks to humans necessitate tougher restrictions, increase liability exposure, and cause reputational harm, thereby decreasing profit margins and discouraging investment throughout the chicken supply or value chain.

## 48. Conclusions

The increasing incidence of antibiotic resistance in poultry production is one of the main concerns for animal and human health, as well as for the environment. The One Health approach recognizes these domains as interrelated and maintains that an evidence-based approach is necessary to tackle this crisis. The progress in the development and use of substitutes for antibiotics, the improvements in existing legislation, and the increase in consumer awareness, as well as international cooperation in reducing the usage of antibiotics, provide opportunities to weaken the dependence on antibiotics without reducing the effectiveness of agricultural production or the stability of food security. Nonetheless, due to the systemic nature of the problem, constant efforts from all stakeholders, including at the governmental, industrial, academic, and consumer levels, are required. They can redirect focus on innovation in poultry education and the fair distribution of resources to ensure a sustainable future for health and biodiversity, which are threatened by the increasing incidence of resistance to antimicrobials.

## Figures and Tables

**Table 1:** Detailed overview of antibiotic use and resistance in poultry farming.

Antibiotic Class	Common Antibiotics Used	Primary Uses in Poultry	Mode of Action	Issues with Resistance	Side Effects in Poultry	Implications for Human Health	References
Tetracyclines	Tetracycline, Doxycycline	Respiratory infections, mycoplasma infections	Inhibits bacterial protein synthesis by binding to 30S ribosomal subunit	Frequent resistance genes reported in the environment	Growth retardation, altered gut microbiota	Increased prevalence of tetracycline-resistant pathogens	[26,27]
Fluoroquinolones	Enrofloxacin, Ciprofloxacin	Respiratory and enteric bacterial infections (e.g., *Escherichia coli*, *Salmonella* spp.)	Inhibits DNA gyrase and topoisomerase IV	Resistance developed due to misuse in treatment	Joint cartilage damage in young birds	Fluoroquinolone-resistant bacteria affecting human therapy	[28]
Sulfonamides	Sulfamethoxazole, Sulfadiazine	*Escherichia coli* infections, Coccidiosis (in combination with trimethoprim)	Inhibits folate synthesis pathway	Ongoing susceptibility issues in food animals	Kidney damage, hypersensitivity reactions	Cross-resistance with human pathogens like Pseudomonas	[29]
Aminoglycosides	Gentamycin, Neomycin	Severe bacterial diseases (e.g., *Escherichia coli*, *Salmonella* spp.)	Binds to 30S ribosomal subunit causing misreading of mRNA	Restricted use due to resistance concerns	Nephrotoxicity, ototoxicity	Aminoglycoside-resistant infections in humans	[30,31]
Polypeptides	Colistin	Control of *Escherichia coli* and *Salmonella* spp. infections	Disrupts bacterial cell membrane	Resistance emerging from excessive feed use	Reduced nutrient absorption, gut toxicity	Emergence of colistin-resistant superbugs	[28]
Beta-Lactams	Amoxicillin, Cephalosporins	Management of respiratory infections and necrotic enteritis (e.g., *Clostridium perfringens*)	Inhibits bacterial cell wall synthesis	Emergence of ESBL-producing bacteria, reducing beta-lactam efficacy	Allergic reactions, gut dysbiosis	ESBL bacteria limiting human antibiotic options	[26,28]

**Table 2:** Overview of antibiotic resistance mechanisms in poultry: biological basis, implications, and prevention strategies.

**Resistance Mechanism**	Description	Mechanism of Action	Key Examples	Examples of Resistant Bacteria	Impact on Poultry Health	Implications for Human Health	Prevention/Mitigation Strategies	Current Challenges	References
Enzyme Degradation	Bacteria produce enzymes that degrade or modify antibiotics, such as beta-lactamases that inactivate beta-lactam antibiotics.	Hydrolysis of antibiotic active sites	Beta-lactamases, aminoglycoside-modifying enzymes	*Escherichia coli*, *Klebsiella pneumoniae*	Decreased antibiotic efficacy, increased disease prevalence	Transmission of resistant pathogens via poultry products	Use of enzyme inhibitors; rotation of antibiotics	Limited availability of enzyme–inhibitor combinations in animals	[26,27,28]
Target Site Alterations	Alterations in the antibiotic binding site, such as mutations in DNA gyrase or methylation of ribosomal RNA, prevent effective antibiotic action.	Modification of binding site for antibiotics	Fluoroquinolone-resistant *Campylobacter jejuni*, macrolide-resistant *Campylobacter jejuni*	*Campylobacter jejuni*, *Salmonella* spp.	Harder to control infections due to resistant strains	Reduced efficacy of critical antibiotics in human medicine	Development of precision diagnostics to detect mutations	Rapid emergence of mutations with antibiotic overuse	[37]
Efflux Pumps	Membrane proteins pump out antibiotics, reducing intracellular concentrations to non-lethal levels, leading to multidrug resistance.	Active expulsion of antibiotics from bacterial cells	Tet(A), Tet(K) in *Escherichia coli*, AcrAB-TolC efflux pump in *Escherichia coli*	*Escherichia coli*, *Pseudomonas aeruginosa*	Multidrug resistance reduces treatment options	Multidrug-resistant bacterial infections in humans	Development of efflux pump inhibitors	High adaptability of efflux pump mechanisms	[38,39,40]
Horizontal Gene Transfer	Transfer of resistance genes between bacteria via plasmids, bacteriophages, or transduction, enabling rapid spread of resistance.	Transfer of genetic material through plasmids, conjugation	mcr-1 gene in *Escherichia coli* and *Salmonella* spp.	*Salmonella, Enterococcus* spp.	Increased spread of resistant bacteria in dense poultry farms	Global dissemination of resistance genes impacting public health	Improved farm hygiene; restriction on antibiotic use	Ineffectiveness in controlling horizontal transfer in farm settings	[41,42]
Biofilm Formation	Protective bacterial films make antimicrobial agents less effective, particularly in managing *Salmonella spp.* and *Campylobacter jejuni* infections.	Formation of extracellular matrix shields	Biofilms in *Salmonella* spp. and *Campylobacter jejuni*	*Salmonella, Listeria monocytogenes*	Persistent infections that are difficult to treat	Chronic infections due to biofilm-protected pathogens	Use of biofilm-disrupting agents; regular sanitization	Difficulty in eradicating established biofilms	[43,44,45]
Reduced Membrane Permeability	Bacteria reduce membrane permeability by downregulating porins, hindering antibiotic access, and leading to resistance.	Decreased porin production; altered membrane fluidity	Beta-lactam and carbapenem resistance in *Escherichia coli*	*Escherichia coli*, *Klebsiella pneumoniae*	Higher mortality and morbidity rates due to resistant strains	Limited options for treating Gram-negative bacterial infections	Monitoring porin expression; use of combination therapies	Difficult detection and monitoring of porin changes	[46,47]
Plasmid-Mediated Resistance	Plasmids carry and disseminate resistance genes, such as mcr-1 for colistin resistance, across bacterial species.	Horizontal spread of plasmids containing resistance genes	Plasmids carrying ESBL or mcr-1 genes	*Escherichia coli*, *Salmonella* spp.	Rapid resistance spread makes disease outbreaks harder to control	Resistance genes spreading to human pathogens, complicating treatments	Limiting plasmid spread through biosecurity measures	Rapid plasmid exchange under high-density farming conditions	[48,49]

## Data Availability

No new data were created or analyzed in this study.
